# Dental Fear Survey: A Cross-Sectional Study Evaluating the Psychometric Properties of the Brazilian Portuguese Version

**DOI:** 10.1155/2014/725323

**Published:** 2014-08-11

**Authors:** Maurício Antônio Oliveira, Miriam Pimenta Vale, Cristiane Baccin Bendo, Saul Martins Paiva, Júnia Maria Serra-Negra

**Affiliations:** Department of Pediatric Dentistry and Orthodontics, School of Dentistry, Universidade Federal de Minas Gerais, Avenida Presidente Antônio Carlos 6627, 31270-901 Belo Horizonte, MG, Brazil, Brazil

## Abstract

*Objective*. The aim of this study was to evaluate the psychometric properties of the Brazilian version of the Dental Fear Survey (DFS), previously translated to the Brazilian Portuguese language and validated. *Methods*. A cross-sectional study with 1,256 undergraduates from the city of Belo Horizonte, Brazil, was carried out. The DFS and a questionnaire about previous dental experiences were self-administered. Data analysis involved descriptive statistics, principal components analysis (PCA), confirmatory factor analysis (CFA), internal consistency and test-retest reliability, and construct, discriminant, and convergent validity. *Results*. PCA identified a three-factor structure. CFA confirmed the multidimensionality of the Brazilian version of the DFS. A modified model of the Brazilian version of the DFS fits better than the hypothesized model. The Cronbach's alpha coefficient for the total DFS scale was 0.95. *Conclusion*. The DFS demonstrated acceptable construct validity, convergent validity, and discriminant validity. These results supported the reliability and validity of the DFS among Brazilian undergraduates.

## 1. Introduction

Despite all the technological advancements in the dental profession, fear toward dentistry remains a major concern and potentially distressing problem in daily practice [1, 2]. Several methods to study dental fear have included the use of questionnaires and behavioural measures and have emphasized the importance of ensuring that such measures are reliable, valid, and applicable to the population toward which they are aimed [1, 3].

Kleinknecht's Dental Fear Survey (DFS) [4] is one of the most frequently used measures of dental fear [1, 3] and has been used in international epidemiological studies for over 30 years [4, 5]. It is a scale used in behavioural research studies, which presents good stability, high reliability, and acceptable validity in diverse cultures and languages [6–14].

The DFS was validated in Brazil about 20 years ago among psychology undergraduates [7]. The original three-factor structure of the DFS was found, suggesting a multidimensionality of the Brazilian version of the DFS [7]. However, internal consistency and discriminant validity were measured only for the DFS total score considering unidimensionality [7]. A construct validity or confirmatory factor analyses (CFA) were not performed [7].

In the last 20 years the validation criteria of instruments have been improved. Given the improvement of psychometric properties' analyses over this time and the lack of testing of the Brazilian version of the DFS as a multidimensional scale, the aim of this study was to confirm the factor structure of the instrument and to evaluate the validity and reliability of the Brazilian version of the DFS scales in a large convenient sample of undergraduates from different fields of study including health (dentistry) and hard (mathematics) and soft (psychology) sciences.

## 2. Materials and Methods

### 2.1. Measure of Dental Fear

The DFS was developed among students (college, high school, and junior high school) from Bellingham, WA, USA [4]. The original DFS contained 27 items to identify specific fear stimuli and measure patients' reactions. The questionnaire assessed items concerning the avoidance of dentistry, physiological arousal during dental appointment, various items of dental stimuli such as seeing the needle, and smelling the smell of the dental office. In addition, one item asks for an overall rating of general fear of dentistry and four items solicit information concerning reactions to dentistry among family and friends [4]. Later, the authors reduced the DFS to 20 items as a result of a factor analysis [5, 15]. The DFS was validated in a study conducted in different samples of dental patients and psychology undergraduates [5].

The current DFS is composed of 20 items, 5-point scales, comprising three dimensions: avoidance (8 items), physiological arousal (5 items), and fears of specific stimuli/situations (7 items). The response options follow a rating scale ranging from “never” or “not at all” (score = 1) to “nearly every time” or “very much” (score = 5). Avoidance scores can range from 8 to 40, physiological arousal from 5 to 25, and fears of specific stimuli/situations from 7 to 35. The total for DFS scores could range from 20 to 100, with highest scores indicating high dental fear [5, 6].

The DFS was cross-culturally adapted and validated among Brazilian psychology undergraduates in the early 1990s [7]. It was translated into the Brazilian Portuguese language by a bilingual native speaker and then backtranslated, pretested, and revised. Factor analysis of the Brazilian version of the DFS demonstrated three consistent factors, which explained 66.3% of the scale variance (8). The Brazilian version of the DFS was self-completed by the undergraduates in this study [7].

### 2.2. Data Collection

A cross-sectional study was carried out at the* Universidade Federal de Minas Gerais* (UFMG) [16] to assess the psychometric properties of the Brazilian version of the DFS. UFMG is located in Belo Horizonte, the capital of the state of* Minas Gerais*, Brazil. Following authorization from the Human Research Ethics Committee of the UFMG, written consents were obtained from the directors of the departments of each course and from the teachers. The undergraduates were invited to participate voluntarily in the study. They were assured that the nonparticipation in the study would not result in a lower mark in their class. After explaining the aim and importance of the study, informed consent forms were signed by the volunteer undergraduates. The researcher remained in the lecture room during data collection.

During the first half of 2010, the questionnaires were tested in a pilot study including 80 students from three courses. These students did not participate in the main study. The results indicated that changes to the proposed methodology were not necessary.

The data of the main study were collected from August to December in 2010 in dentistry, psychology, and mathematics colleges. All the students enrolled in the three undergraduate courses were included, from the first to the last year of the course, totaling 1,565 individuals. The students self-completed the Brazilian version of the DFS, as well as a pretested questionnaire used to collect data relating to dental experience. A total of 1,256 individuals participated in the study, with a response rate of 80.25%. The main reasons for the nonresponse rate were the refusal of seven students (0.45%) and the absence of 302 students (19.30%) in the days for data collection [17].

## 3. Statistical Analysis

The Statistical Package for the Social Sciences (SPSS for Windows, version 19.0, SPSS Inc., Chicago, IL, USA) was used for statistical analysis. Data analysis involved descriptive statistics such as frequencies, ceiling and floor effects, means, and standard deviations (SD). Mann-Whitney test was used to compare DFS scales scores regarding gender. Acceptable floor or ceiling effects are less than or equal to 15% [18]. The Kolmogorov-Smirnov test demonstrated that the data had a nonnormal distribution.

### 3.1. Principal Components Analysis

Principal components analysis (PCA) was used to evaluate the items and determine if the structure of the scale was the same as the original USA version [5]. PCA was conducted based on the extraction of factors by principal components analysis and Eigenvalues of greater than one were used to determine the number of factors. The Promax method was used for rotation. Factor loadings equal to or greater than 0.4 were considered adequate [19].

### 3.2. Confirmatory Factor Analysis

Confirmatory factor analysis (CFA) was conducted to test model fit of a priori model and confirm the multidimensionality of the Brazilian version of the DFS. The LISREL for Windows software (version 8.8, Scientific Software International Inc., Lincolnwood, IL, USA) was used for this analysis. To unstandardized solutions, the pattern of fixed and free-factor loadings was held constant. The chi-square (*χ* ^2^) statistic is an extremely sensitive statistical test, not interpretable in a standardized way and not a practical test of model fit [19]. According to the recommendations in the existing literature [20, 21] other model indices of practical fit can be used including the normed fit index (NFI), comparative fit index (CFI), goodness of fit index (GFI), and root mean squared error of approximation (RMSEA). For the NFI, CFI, and GFI indices, excellent model fit is suggested by values greater than or equal to 0.95, while acceptable model fit is suggested by values between 0.90 and 0.95. Excellent model fit is suggested by RMSEA values less than or equal to 0.06, while acceptable model fit is suggested by RMSEA values between 0.06 and 0.08 [22].

### 3.3. Internal Consistency Reliability

The internal consistency reliability of the Brazilian version of the DFS was estimated using Cronbach's alpha coefficient. Values ≥ 0.70 are considered acceptable [19, 23].

### 3.4. Test-Retest Reliability

Intraclass correlation coefficient (ICC) for test-retest reliability of the DFS was obtained on two occasions, separated by an interval of two weeks with 80 undergraduates. The ICC provides an index of absolute agreement as it takes into account the ratio between subject variability and total variability andcounts the repetition variability as a more stringent criterion [24]. ICCs < 0.40 show poor to fair agreement, 0.41–0.60 show moderate agreement, 0.61–0.80 show good agreement, and show >0.80 excellent agreement [25].

### 3.5. Construct Validity

The construct validity was determined by calculation of the intercorrelations among the scores of the three factors and total scale of the Brazilian version of the DFS. Spearman's correlation coefficient was calculated.

### 3.6. Convergent Validity

Correlational analysis was also conducted to assess the convergent validity between the scores of the DFS and self-report dental fear using the global question “do you fear going the dentist's office?” Correlation coefficients are designated as small (0.10–0.29), medium (0.30–0.49), and large (>0.50) [26].

### 3.7. Discriminant Validity

The sensitivity of the Brazilian version of the DFS may be demonstrated through the discriminant validity. A comparison of means and SD between undergraduates who had negative dental experiences in childhood and those did not was conducted. Mann-Whitney test was used for this analysis. We hypothesized that the DFS would distinguish between those who reported negative dental experiences in childhood and those who did not report such experiences. Effect sizes were calculated to determine the magnitude of the differences, and differences in means are designated as small (0.20), medium (0.50), and large (0.80) in magnitude [26].

## 4. Results

### 4.1. Descriptive Analysis

The DFS was completed by 1,256 dentistry, psychology, and mathematics undergraduates. The students' ages ranged from 18 to 65 years, with a mean of 22.3 years (SD = 5.1). There was a predominance of females (62.9%) over males (37.1%) in the total sample. The mean of the DFS total scale was 34.8 (SD = 13.1). Females were more fearful than males in the fears of specific stimuli/situations scale (mean = 16.2, SD = 7.0 versus mean = 14.7, SD = 6.6; *P* < 0.001), avoidance scale (mean = 11.3, SD = 4.6 versus mean = 10.9, SD = 4.5; *P* = 0.034), and DFS total (mean = 35.6, SD = 13.4 versus mean = 33.5, SD = 12.6; *P* = 0.002).

Table 1 shows the percentage of scores at the extremes of the scaling range (floor and ceiling effects) for the Brazilian version of the DFS. There were no significant floor effects for fears of specific stimuli/situations and DFS total scale. Ceiling effects were not detected.

### 4.2. Reliability

Table 1 shows the internal consistency reliability values estimated by Cronbach's alpha coefficient. The values ranged from 0.86 to 0.95, exceeding the minimum reliability standard of 0.70, indicating adequate internal consistency. ICC results were 0.882 (95% CI: 0.793–0.930) for avoidance, 0.874 (95% CI: 0.810–0.917) for physiological arousal, and 0.897 (95% CI: 0.829–0.937) for fears of specific stimuli/situations. This data confirmed the excellent stability of the DFS factors.

### 4.3. Principal Components Analysis (PCA)

The PCA led to three structural constructs, and the loading between factors and items is reported in Table 2. The three factors with Eigenvalues exceeding one, based on principal components analysis and the Promax method for rotation, explained 66.2% of the scale's total variance. The first factor (Factor I) was related to fear of specific dental stimuli (items 14–20) and accounted for 51.5% of the scale's total variance. The second factor (Factor II) was related to patterns of dental avoidance and anticipatory anxiety (items 1, 2, and 8–13) and accounted for 8.0% of the scale's total variance. The third factor (Factor III) was related to physiological arousal during dental treatment (items 3–7) and accounted for 6.7% of the scale's total variance.

### 4.4. Confirmatory Factor Analysis (CFA)

The multidimensionality of the Brazilian version of the DFS was confirmed. A priori hypothesized model, with each of the 20 items of the instrument loading in only one factor, did not fit the data well. The goodness of fit statistics were NFI = 0.95, CFI = 0.95, GFI = 0.80, and RMSEA = 0.12. In order to improve the overall model fit, error covariance was added between DFS 1 and DFS 2, DFS 6 and DFS 12, DFS 8 and DFS 9, DFS 9 and DFS 10, DFS 14 and DFS 15, and DFS 18 and DFS 19. Paths from Factor II to DFS 14, DFS 19, and DFS 20 were included. A path from Factor III to DFS 20 was also added. The goodness of fit indices for modified model were NFI = 0.98, CFI = 0.98, GFI = 0.91, and RMSEA = 0.07.

### 4.5. Validity

The intercorrelations between the three factors and total scale scores of the Brazilian version of the DFS were determined by Spearman's correlation coefficient in order to determine construct validity. These are shown in Table 3. All correlations were significant at the 0.001 level and can be classified as large. The convergent validity was obtained comparing scores between DFS scales and a global question about dental fear. The Spearman correlation coefficients (*r*) were 0.640 for DFS total, 0.626 for avoidance, 0.540 for physiological arousal, and 0.576 for fears of specific stimuli/situations scale. All the correlations were statistically significant at 0.001 levels.

Table 4 presents the differences between the undergraduates with and without negative dental experiences in childhood for the Brazilian version of the DFS factors and total scale. Undergraduates who had negative dental experiences in childhood reported statistically significantly higher dental fear (higher DFS scores) than those who did not report such experiences (*P* < 0.001).

## 5. Discussion 

The present study supports the validity and reliability of the Brazilian version of the DFS, and three structural constructs were identified. Factor I refers to fears of specific stimuli/situations (DFS items 14 to 20), Factor II refers to avoidance (DFS items 1, 2, and 8–13), and Factor III refers to physiological arousal (DFS items 3–7). The distribution of the items in the three factors, considering the loadings of PCA, differed slightly from a previous Brazilian version of the DFS [7].

The previous study affirmed that DFS item number 12 had stronger loading in fears of specific stimuli/situations factor instead of avoidance factor [7]. However, this difference could be considered small, since the loadings between DFS item number 12 and both factors were above 0.4 [7].

The three structural factors explained 66.2% of the scale's total variance in this present study. However, the first factor (fears of specific stimuli/situations) was almost twice as much of the scale's total variance (51.5%) compared to the result of the Brazilian validation study (27.3%) [7]. In contrast, the second (avoidance) and third factors (physiological arousal) both accounted for about a three times lower percentage of the scale's variance in this study (8.0% and 6.7%) compared with the previous study (21.7% and 17.3%, resp.) [7]. The distribution of the items forming these factors was little different from the original DFS and other validated versions of the DFS [5–7, 12] but was exactly the same as the Japanese version of the DFS [10].

Therefore, a major concern is the lack of clarification about distributions of the items according to factors [5–7, 10]. Most of these previous studies did not clarify the factor structure of the DFS, leaving the readers uncertain about it, which may cause misunderstanding [5–7]. A modified model fit to the Brazilian version of the DFS showed logical paths between items and the three latent factors, with squared standardized factor loadings demonstrating that each factor was able to explain the variances in manifest variables (items of the DFS). This modified model fit well and demonstrated an improvement in fit statistics compared with the hypothetical model. This hypothetical model was similar to the factor structure of the Japanese version of the DFS [10], but the correlations between latent variables were different in the Japanese study, perhaps due to study design and cultural differences between the populations. In contrast, Sirin et al. [12] considered a new distribution pattern of 18 items only instead of 20 items, which resulted in a better statistical fit to reliable evaluation of fear in oral surgery patients.

One of the modifications in the model is relating to DFS item number 20. This item is an overall summary item. Even though item number 20 loaded stronger in fear of specific stimuli/situations factor (Factor I) when the PCA was conducted, the CFA demonstrated that this item is also explained for avoidance (Factor II) and physiological arousal (Factor III) factors. A similar finding was reported by previous studies [5, 6, 10].

High internal consistency was revealed by Cronbach's alpha coefficient. The coefficient for the DFS total scale was 0.95, exactly the same as found by Cesar et al. [7] and Sirin et al. [12]. The three factors showed coefficients above 0.80, demonstrating excellent internal consistency. The excellent ICC obtained for this sample demonstrated that the three factors of the Brazilian version of the DFS are highly reproducible.

The construct validity of the Brazilian version of the DFS in the study was evidenced by the significant intercorrelations between the three factors and total scale scores and was classified as large. According to the existing literature, computing the intercorrelations among scales provides initial information on the construct validity of an instrument [27]. Evidence of discriminant validity was supported by the ability of the Brazilian version of the DFS to discriminate between groups with and without negative dental experiences in childhood. This result is consistent with the literature, which found that negative dental experience in childhood could significantly predict the persistence of high dental fear in adults [2, 4, 11, 12, 14, 28, 29].

Convergent validity was supported by a comparison of the Brazilian version of the DFS and a global question about dental fear. Although there are many and complex components of fear [3] the three factors of the DFS seem to be adequate to measure what they attempt to measure. However, this result reflects the general dental fear of undergraduates investigated during lecture classes and could be different in a clinical setting. This reflection is consistent with the analysis about the theoretical construct of the DFS which is a measure that could help understand patients' fear but is not a measure to understand the fear as a complex emotion [3].

The sample was composed exclusively of undergraduates, most of whom were females, which may inflate the DFS scores over those that might be seen in the general population, since females often report being more fearful than males [4, 9, 11, 14, 30]. Moreover, the relationships between the three factors of the DFS might be different in a clinical sample. These limitations may minimize the generalizability of the results. Therefore, the use of a self-reported questionnaire could be affected by attention or recall bias [31]. It would be desirable to replicate the results using other samples from Brazilian populations. However, further studies may be helpful in this regard.

Despite all the technological advancements in the dental profession, dental fear remains associated with previous negative experiences. Four decades ago, adverse reactions to the dentist's office were often attributed to a personal dislike of their dentist or fear of needle and drill [4]. The ability of a dentist to relieve behavioural problems and dental fear was also reported as predictors of dental fear [2, 13, 32, 33]. In the present study, the intensity of the fear was based on the sum of DFS scores, but cultural differences and interpretations of the items by subjects can result in different conclusions [12].

## 6. Conclusions

The Brazilian version of the DFS demonstrated acceptable reliability and validity, thereby confirming the applicability of this instrument among Brazilian undergraduates. The psychometric properties were found to be satisfactory. The analysis of the DFS factor “fears of specific stimuli/situations” showed a significant association with “negative dental experiences in childhood.” The DFS is an informative measure that could help dentists better understand a patient's fear during dental treatment.

## Supplementary Material

The Brazilian version of the DFS is composed of 20 items divided into three dimensions. The items #1, 2, 8 to 13 pertain to the Avoidance dimension. The items #3 to 7 compose the Physiological arousal dimension. The Fears of specific stimuli/situations dimension is composed for items #14 to 20. The response option follow a rating scale ranging "never" or "not at all" to "nearly every time" or "very much", with highest scores indicating high dental fear.

## Figures and Tables

**Table 1:** Descriptive analysis and Cronbach's alpha (*α*) coefficient for the Brazilian version of the Dental Fear Survey (DFS) (*n* = 1,256)

Scale	Number of items	Mean (SD)	Percent floor (%)	Percent ceiling (%)	Cronbach's *α*
DFS total	20	34.83 (13.10)	7.2	0.1	0.95
Avoidance	8	11.18 (4.54)	34.9	0.1	0.90
Physiological arousal	5	8.00 (3.20)	25.6	0.2	0.86
Fears of specific stimuli/situations	7	15.65 (6.89)	10.6	0.6	0.93

SD = standard deviation.

**Table 2:** Structure loadings for the three-factor solution with Promax rotation and correlations of the Dental Fear Survey (DFS) with each item.

Item	Pattern matrix factor loadings		
	Factor I	Factor II	Factor III
Avoidance			
DFS1. Has fear of dental work ever caused you to put off making an appointment?	0.022	0.640	0.068
DFS2. Has fear of dental work ever caused you to cancel or not appear for an appointment?	−0.149	0.766	−0.062
DFS8. Making an appointment for dentistry	0.013	0.908	−0.220
DFS9. Approaching the dentist's office	−0.078	0.886	0.029
DFS10. Sitting in the waiting room	−0.016	0.752	0.093
DFS11. Being seated in the dental chair	0.289	0.445	0.242
DFS12. The smell of the dentist's office	0.303	0.419	0.169
DFS13. Seeing the dentist walk in	0.165	0.600	0.143

Physiological arousal. *When having dental work done*:			
DFS3. My muscles become tense	0.223	−0.108	0.753
DFS4. My breathing rate increases	0.016	−0.107	0.940
DFS5. I perspire	−0.104	−0.034	0.904
DFS6. I feel nauseated and sick to my stomach	−0.131	0.191	0.558
DFS7. My heart beats faster	−0.042	0.008	0.889

Fears of specific stimuli/situations			
DFS14. Seeing the anesthetic needle	0.801	−0.111	0.121
DFS15. Feeling the needle injected	0.901	−0.186	0.074
DFS16. Seeing the drill	0.958	0.019	−0.108
DFS17. Hearing the drill	0.965	0.032	−0.126
DFS18. Feeling the vibrations of the drill	0.985	−0.038	−0.114
DFS19. Having your teeth cleaned	0.600	0.111	0.012
DFS20. All things considered, how fearful are you of having dental work done?	0.531	0.222	0.209

**Table 3:** Construct validity: rank correlations between scores of total scale and factors of the Brazilian version of the Dental Fear Survey (DFS) (*n* = 1,256).

Scale	DFS	Avoidance	Physiological arousal	Fears of specific stimuli/situations
DFS total	—			
Avoidance	0.840∗	—		
Physiological arousal	0.785∗	0.637∗	—	
Fears of specific stimuli/situations	0.954∗	0.711∗	0.629∗	—

Categories of the correlations are small (0.10), medium (0.30), and large (0.50).

Spearman's correlation coefficient; *P < 0.001.

**Table 4:** Discriminant validity of the Brazilian version of the Dental Fear Survey (DFS) total scale and factors according to negative dental experiences in childhood (*n* = 1,255).

	Negative dental experiences in childhood		*P* value∗	Difference	Effect size
	No (*n* = 961) Mean (SD)	Yes (*n* = 294) Mean (SD)			
DFS total	32.62 (11.39)	42.04 (15.56)	<0.001	9.426	0.70
Avoidance	10.45 (3.72)	13.56 (5.96)	<0.001	3.104	0.64
Physiological arousal	7.58 (2.79)	9.36 (3.98)	<0.001	1.776	0.52
Fears of specific stimuli/situations	14.58 (6.33)	19.13 (7.50)	<0.001	4.545	0.66

SD = standard deviation.

Mann-Whitney test.
