# Supplementary material for: Dental Fear Survey: A Cross-Sectional Study Evaluating the Psychometric Properties of the Brazilian Portuguese Version
Source: ScientificWorldJournal. 2014 Aug 11;2014:725323. doi: 10.1155/2014/725323 (PMC4144391; doi:10.1155/2014/725323)
Supplement: Supplementary file 1 — The Brazilian version of the DFS is composed of 20 items divided into three dimensions. The items #1, 2, 8 to 13 pertain to the Avoidance dimension. The items #3 to 7 compose the Physiological arousal dimension. The Fears of specific stimuli/situations dimension is composed for items #14 to 20. The response option follow a rating scale ranging "never" or "not at all" to "nearly every time" or "very much", with highest scores indicating high dental fear. [file 725323.f1.pdf]

Additional File 1

The Brazilian Portuguese version of the Dental Fear Survey (DFS)

Os itens neste questionário se referem a várias situações, sentimentos e reações relacionadas com o tratamento dentário. Por favor, avalie seu sentimento ou reação com relação a estes itens fazendo um círculo ao redor do número (1, 2, 3, 4 ou 5) da categoria que melhor corresponde a sua reação.

Nome: ______________________ Data: ____/____/____

Sexo: Feminino ( ) Masculino ( ) Idade: ______________________

Profissão do Pai: ______________ Profissão da Mãe: ______________

Que escola cursou anteriormente? ( ) Particular ( ) Pública

Quando foi sua última visita ao dentista? ( ) 6 meses ( ) 1 ano ( ) 2 anos ( ) 3 anos ( ) outros

Qual o tipo de tratamento realizado na última visita?

( ) Exame clínico ( ) Restauração ( ) Profilaxia ( ) Extração

( ) Tratamento de canal ( ) Alívio de dor ( ) Outros

1. O medo do tratamento dentário já levou você a demorar a (adiar) marcar consulta?

1	2	3	4	5
Nunca	Uma ou duas vezes	Algumas vezes	Frequen-temente	Quase sempre

2. O medo do tratamento dentário já levou você a cancelar uma consulta ou simplesmente não comparecer?

1	2	3	4	5
Nunca	Uma ou duas vezes	Algumas vezes	Frequen-temente	Quase sempre

3. Quando me submeto ao tratamento dentário: meus músculos ficam tensos

1	2	3	4	5
Nem um pouco	Um pouco	Mais ou menos	Muito	Muitíssimo

4. O ritmo da minha respiração aumenta

1	2	3	4	5
Nem um pouco	Um pouco	Mais ou menos	Muito	Muitíssimo

5. Eu transpiro

1	2	3	4	5
Nem um pouco	Um pouco	Mais ou menos	Muito	Muitíssimo

6. Sinto náuseas e enjôo de estômago

1	2	3	4	5
Nem um pouco	Um pouco	Mais ou menos	Muit o	Muitíssimo

7. Meu coração bate mais depressa

1	2	3	4	5
Nem um pouco	Um pouco	Mais ou menos	Muito	Muitíssimo

A seguir há uma lista de coisas e situações que muitas pessoas mencionam como sendo algo como ansiedade ou medo. Por favor, avalie quanto medo, ansiedade ou desprazer cada uma delas causa em você. Use os números de 1 a 5 da escala a seguir. Faça uma marca no espaço apropriado (se ajudar, tente imaginar você mesmo em cada uma destas situações e descreva qual é a sua reação habitual).

1	2	3	4	5
Nem um pouco	Um pouco	Mais ou menos	Muito	Muitíssimo

	1	2	3	4	5
8. Marcando consulta para ir ao dentista					
9. Aproximando-se do consultório do dentista					
10. Aguardando na sala de espera					
11. Estar sentado na cadeira do dentista					
12. Sentindo o cheiro do consultório					
13. Vendo o dentista entrar					
14. Vendo a agulha da seringa					
15. Sentindo a agulha penetrar					
16. Vendo a broca do motor					
17. Ouvindo o motor					
18. Sentindo as vibrações do motor no dente					
19. Submetendo-se à limpeza dos dentes					
20. Considerando todas as situações, quanto medo você sente em relação ao tratamento dentário?					

Note: The DFS was previously translated and validated by Cesar et al. (1993).
